# Prevalence and predictors of antidepressant use in a cohort of pregnant women

**DOI:** 10.1111/j.1471-0528.2007.01387.x

**Published:** 2007-06-12

**Authors:** É Ramos, D Oraichi, É Rey, L Blais, A Bérard

**Affiliations:** a Research Center, CHU Sainte-Justine Hospital Montreal, Quebec, Canada; b Department of Obstetrics & Gynaecology, CHU Sainte-Justine Hospital Montreal, Quebec, Canada; c Research Center, Sacré-Coeur Hospital Montreal, Quebec, Canada

**Keywords:** Antidepressants, dosage, predictors, pregnancy, prevalence, switch

## Abstract

**Objective:**

(1) To determine the prevalence of antidepressant utilisation before, during, and after pregnancy, (2) to determine switches, dosages, and classes of antidepressant used during pregnancy, and (3) to identify factors associated with their use at the beginning and at the end of pregnancy.

**Design:**

Retrospective longitudinal cohort.

**Setting:**

The ‘Medication and Pregnancy’ cohort was used for this study. This cohort was built by the linkage of three administrative databases (Régie de l’Assurance Maladie du Québec [RAMQ], Med-Écho, and l’Institut de la Statistique du Québec).

**Population:**

All pregnancies occuring in Quebec between January 1 1998 and December 31 2002.

**Methods:**

Date of entry in the cohort was the first day of gestation. To be eligible for this study, women had to be (1) 15–45 years old at cohort entry and (2) covered by the RAMQ drug plan for at least 12 months before, during, and at least 12 months after pregnancy. Antidepressant users were defined as those receiving at least one antidepressant before, during, or after pregnancy, depending on the time period analysed. Logistic regression models were used to identify factors associated with receiving an antidepressant either at the beginning or at the end of pregnancy.

**Main outcome measures:**

To determine the prevalence and predictors associated with the use of antidepressants.

**Results:**

A total of 97 680 women met inclusion criteria. The prevalence rates significantly declined during the first trimester compared with before pregnancy (3.7 versus 6.6%, *P* < 0.01). During pregnancy, antidepressants were used under the recommended daily dosage 7.7% of the time, and 4.7% of women switched to another class of antidepressant. Factors significantly associated with antidepressant utilisation on the first day of gestation (*P* < 0.05) were older maternal age, being on welfare, and calendar year; receiving at least six different types of medications other than antidepressants, having at least two different prescribers, having at least three visits to the physician, and having at least one diagnosis of depression in the year before pregnancy also increased the odds of having an antidepressant. Similar predictors were found at the end of pregnancy.

**Conclusions:**

Our findings indicate that antidepressant utilisation declines once pregnancy is diagnosed.

*Please cite this paper as:* Ramos É, Oraichi D, Rey É, Blais L, Bérard A. Prevalence and predictors of antidepressant use in a cohort of pregnant women. BJOG 2007;114:1055–1064.

## Introduction

Depression is a common disorder in women of childbearing age. Indeed, up to 9% of women experience depression during pregnancy.^1^,^2^ Nevertheless, antidepressant use during the gestational period remains a controversial topic. One report on mental health suggests that physicians may often underprescribe or stop antidepressants at the time of conception and during pregnancy.^3^ This may be a consequence of the concern over the safety of these agents in pregnant women and the risks they may pose to the fetus. In fact, since the thalidomide disaster in 1962, antidepressants like other medications used during pregnancy have often been associated in the mind of the public with congenital malformations or other severe complications in newborns without any scientific evidence.^4^,^5^ The baseline prevalence of major malformation is known to be 1.5–3%.^6^ Therefore, a substantial number of children could be born with some birth defects without the influence of maternal exposure to medications. This notion may be frequently misunderstood by health providers and the general population. However, there are reports of antidepressant drug use and teratogenic effects, for example recent associations between first-trimester exposure to selective serotonin reuptake inhibitors (SSRIs) and cardiac malformations or persistent pulmonary hypertension of the newborn, which have raised justifiable concerns^7^,^8^ that require further study.

Discontinuation of antidepressant use during pregnancy is also associated with relapse of depression and withdrawal symptoms, which is not optimal for the mother and her fetus^9^. Even after reinstating the antidepressant, it may take several weeks for the depression to be controlled.^10^ Furthermore, during pregnancy, psychological stress has been associated with poor perinatal outcomes.^11^,^12^ Therefore, it seems critical enough for clinicians treating women of childbearing age to have information available, which may guide them in treatment decisions during pregnancy, as they negotiate the delicate balance between the use of medications, such as psychotropics and the risks of undertreating depressive disorders.^13^

To our knowledge, there have been no studies that have specifically investigated the prevalence and trends of antidepressant use, before, during, and after pregnancy. Therefore, the purpose of this study was: (1) to determine the prevalence of antidepressant use before, during, and after pregnancy, (2) to determine the percentage of switches during the first trimester of pregnancy, to list the dosages and classes of antidepressant used during pregnancy, and (3) to identify predictors associated with antidepressant use on the first day of gestation and at the end of pregnancy.

## Methods

We used three administrative databases of the Province of Quebec: la Régie de l’Assurance Maladie du Québec (RAMQ), Med-Écho, and Le fichier des événements démographiques du Québec (birth and death registries) of l’Institut de la Statistique du Québec (ISQ). The RAMQ database contains information on medical services (diagnoses and procedures) received by all Quebec residents. All diagnoses are classified according to the *International Classification of Diseases, Ninth Revision (ICD-9)*.^14^ Although RAMQ covers all Quebec residents for the cost of physician visits, hospitalisations, and procedures, it only covers a portion of residents for the cost of medications. The RAMQ drug plan covers individuals 65 years and older, recipients of social assistance (welfare recipients), and workers and their families (adherents) who do not have access to a private drug insurance programme, accounting for approximately 43% of the overall Quebec population.^15^ It is also estimated that 30% of women between 15 and 45 years of age in Quebec are covered by the RAMQ drug plan for their medication (RAMQ data). The Med-Écho database is a provincial database, which records acute care hospitalisation data for all Quebec residents; it also records gestational age for planned abortions, miscarriages, and deliveries. ISQ provides demographic information on the mother, father, and baby, as well as birthweight and gestational age for live births and stillbirths.

The RAMQ and Med-Écho databases have often been used in the past for epidemiological research.^16^–^18^ The ISQ database has also been used in epidemiological studies.^19^ Data recorded in the medication database of the RAMQ have been suitably evaluated and found to be comprehensive and valid.^20^ The same was found for medical diagnoses recorded in the Med-Écho database.^21^

The RAMQ, Med-Écho, and ISQ databases were linked together to create the ‘Medications and Pregnancy’ cohort which contains data on all pregnancies that occurred in Quebec between 1 January 1998 and 31 December 2002. The linkage between the three databases was performed using women's ‘Numero d’Assurance Maladie’, which is the unique identifier for all Quebec residents (RAMQ and Med-Écho), and using mothers and babies dates of birth, first names, and family names (RAMQ and ISQ). This cohort is composed of women with a diagnosis or procedure code related to pregnancy.

Within the ‘Medications and Pregnancy’ cohort, women meeting the following eligibility criteria were included in this study: they had to be (1) between 15 and 45 years of age on the date of entry in the cohort defined as the first day of gestation and (2) continuously insured by the RAMQ drug plan for at least 12 months before the first day of gestation, during the pregnancy, and for at least 12 months after the end of pregnancy. The end of pregnancy was defined as the calendar date of a planned abortion, miscarriage, or delivery. If a woman had more than one pregnancy between 1998 and 2002, the first pregnancy meeting eligibility criteria was included for analysis.

The prevalence of antidepressant use during the ‘12 months before pregnancy’ was calculated by dividing the number of women receiving at least one antidepressant in this 12-month period by the total number of women in the cohort. The same calculations were performed for the time period ‘during pregnancy’ and for the time period ‘12 months after the end of pregnancy’. In addition, the prevalence of antidepressant use in the first trimester (≤14 weeks of gestational age), second trimester (>14 to ≤26 weeks of gestational age), and third trimester (>26 weeks of gestational age) of pregnancy was calculated by dividing the number of women filling at least one antidepressant prescription in the respective trimesters by the number of women in the study at that time (depending on the outcome of the pregnancy, some women were counted in the denominator only in the first or second trimester). When the duration of a prescription overlapped between trimesters, women were defined as exposed in both time periods. The prevalence of antidepressant use according to pharmacological class and type stratified by trimester was calculated in the same manner.

For each dispensed prescription of an antidepressant, the daily dosage was calculated. This daily dosage was then compared with the optimal range recommended by *The Public Health Agency of Canada*.^22^ Since lower dosages are often prescribed at the initiation of a treatment and thus could decrease the overall daily dosage, we used the lower range of what is considered the minimum threshold of pharmacological efficacy.^22^ The daily dosage for each prescription could fall into one of the following three categories: optimal dosage, underdosage, or overdosage. For a given antidepressant, the percentage of prescriptions with optimal dosage was calculated by dividing all prescriptions that were optimally prescribed by the total number of prescriptions. The same calculation was performed to determine the percentage of prescriptions with sub-dosage and overdosage. We restricted these calculations to women with at least one diagnosis of major depressive disorder (ICD-9 codes: 296.2 and 296.3)^14^ either during pregnancy or during the 12 months before pregnancy. This was necessary since these dosage recommendations are intended for women with major depressive disorders.

We determined the percentage of subjects who had at least one switch from one class or type of antidepressant to another in the first trimester.

Women were considered exposed to antidepressants on the first day of gestation and at the end of pregnancy if they filled a prescription or if the duration of a prescription overlapped on these days. In addition, we allowed a 7-day grace period between consecutive prescriptions of antidepressants, and thus, women were considered exposed if the first day of gestation or end of pregnancy fell during this grace period.

The following variables were considered as potential predictors of receiving at least one antidepressant on the first day of gestation and were measured on this day: maternal age, maternal place of residence (urban versus rural), maternal RAMQ drug plan status (adherent versus welfare recipient), and calendar year. The following variables were also considered as potential predictors of receiving at least one antidepressant on the first day of gestation and were measured in the year before pregnancy: number of different types of medications used other than antidepressants, number of different prescribers for all medications, planned abortions or miscarriages, number of visits to the physician, visits to the emergency department and/or hospitalisations, and diagnosis of depression (ICD-9 codes: 296.x; 300.4; 309; 311).^23^

Predictors of antidepressant use at the end of pregnancy were determined only for women who had delivered a baby (live birth and stillbirth) and thus excluded for women whose pregnancies resulted in planned abortions or miscarriages.

As our cohort spanned a 5-year period, we controlled for any time trend in prescribing practices by adjusting the estimates for the calendar year on the first day of gestation.

This study was approved by the Sainte-Justine Hospital Ethics Committee, and by the Commission d’Accès à l’Information du Québec, the agency granting ethics clearance for the use of linked administrative data.

### Statistical analysis

Descriptive statistics were used to summarise the characteristics of the study population. McNemar's test was used to compare the prevalence of antidepressant use before versus during the first trimester of pregnancy and before versus after pregnancy. Unconditional logistic regression models were performed to identify and quantify predictors of antidepressant use on the first day and on the last day of gestation, separately. All analyses were two-tailed, and *P*≤ 0.05 was considered significant. SAS version 8.2 (SAS Institute Inc., Cary, NC, USA) was used to perform the analyses.

## Results

Of the 152 107 women in the ‘Medication and Pregnancy’ cohort with pregnancies between 1 January 1998 and 31 December 2002, a total of 97 680 met the eligibility criteria and were included in the study. The mean (SD) age of the cohort was 27.4 (6.1) years, and the majority of women were adherents of the RAMQ drug plan (65.0%) and urban dwellers (79.9%) on the first day of gestation.

Of the 97 680 pregnancies, 56 981 (58.3%) ended with a delivery, whereas the remaining 40 699 (41.7%) resulted in planned abortions (36 015 [36.9%]) or miscarriages (4684 [4.8%]). Women who had a pregnancy that ended in a delivery, compared with women who had a planned abortion or had a miscarriage were older (28.2 [5.6] years versus 27.6 [6.7] years, *P* < 0.01), were more likely to be adherents of the RAMQ drug plan (68.7 versus 59.0%, *P* < 0.01) and were less likely to be living in urban areas (76.6 versus 84.6%, *P* < 0.01).

The prevalence of antidepressant use during the 12 months before the first day of gestation and during the 12 months after the end of pregnancy was 6.6 and 7.0%, respectively (*P* < 0.01). Rates of antidepressant use declined significantly during the first trimester compared with before pregnancy (3. 7 versus 6.6%, *P* < 0.01) and continued to decrease during the second (1.6%) and the third trimesters (1.1%) (Table 1).

**Table 1:** Prevalence of antidepressant utilisation before, during, and after pregnancy

Period	Number of antidepressant users	Total number of women*	Percent (95% CI)
**During the 12 months before the first day of gestation**	6427	97 680	6.6 (6.4–6.7)
**During pregnancy**			
First trimester (≤14 weeks)	3587	97 680	3.7 (3.6–3.8)**
Second trimester (>14 to ≤26 weeks)	1256	80 164	1.6 (1.5–1.6)
Third trimester (>27 weeks)	618	56 578	1.1 (1.0–1.2)
During the 12 months after the end of pregnancy***	6816	97 680	7.0 (6.8–7.1)****

* Depending on the gestational age at the end of pregnancy, some women were not included in the denominators for the prevalence of use in the second or third trimesters.

** *P* = 0.01—Comparing the 12 months before the first day of gestation to first trimester.

*** The end of pregnancy was defined as a planned abortion, a miscarriage, or a delivery.

**** *P* = 0.01—Comparing the 12 months before the first day of gestation to the 12 months after the end of pregnancy.

During the first trimester of pregnancy, among antidepressant users (*n* = 3587), the three most prevalent antidepressant classes used were SSRIs (2607 [64.4%]), serotonin and norepinephrine reuptake inhibitors (SNRIs) (497 [12.3%]), and tricyclics (491 [12.1%]) (Table 2). More specifically, paroxetine (1385 [34.2%]) and sertraline (515 [12.7%]) were the most frequently used SSRIs, whereas venlafaxine (497 [12.3%]) was the most frequently used SNRI, and amitriptyline (349 [8.6%]) was the most frequently used tricyclic (Table 2). The use of nonhydrazine reversible monoamine oxidase inhibitor (MAOIs) was minimal (3 [0.1%]) (Table 2).

**Table 2:** Class and type of antidepressants used during the first, second, and third trimesters

	First trimester (≤14 weeks) (*n* = 3587)*	Second trimester (>14 to ≤26 weeks) (*n* = 1256)*	Third trimester (>26 weeks) (*n* = 618)*
**SSRIs, *n* (%)**			
Citalopram	304 (7.5)	103 (7.5)	39 (6.0)
Fluoxetine	300 (7.4)	118 (8.6)	64 (9.9)
Fluvoxamine	103 (2.6)	38 (2.8)	20 (3.1)
Paroxetine	1385 (34.2)	504 (36.8)	270 (41.7)
Sertraline	515 (12.7)	180 (13.2)	87 (13.5)
Total	2607 (64.4)	943 (68.9)	480 (74.2)
**Tricyclics, *n* (%)**			
Amitriptyline	349 (8.6)	87 (6.4)	42 (6.5)
Clomipramine	23 (0.6)	7 (0.5)	2 (0.3)
Desipramine	21 (0.5)	10 (0.7)	5 (0.8)
Doxepin	47 (1.2)	19 (1.4)	11 (1.7)
Imipramine	17 (0.4)	9 (0.7)	6 (0.9)
Nortriptyline	18 (0.4)	6 (0.4)	3 (0.5)
Trimipramine	16 (0.4)	7 (0.5)	3 (0.5)
Total	491 (12.1)	145 (10.6)	72 (11.2)
**Tetracyclics, *n* (%)**			
Amoxapine	0 (0.0)	0 (0.0)	0 (0.0)
Maprotiline	1 (0.0)	0 (0.0)	0 (0.0)
Total	1 (0.0)	0 (0.0)	0 (0.0)
**MAOIs, *n* (%)**			
Phenelzine	2 (0.1)	0 (0.0)	0 (0.0)
Tranylcypromine	1 (0.0)	0 (0.0)	0 (0.0)
Total	3 (0.1)	0 (0.0)	0 (0.0)
**Reversible inhibitors of monoamine oxidase type A, *n* (%)**			
Moclobemide	11 (0.3)	1 (0.1)	0 (0.0)
Total	11 (0.3)	1 (0.1)	0 (0.0)
**Dopamine and norepinephrine reuptake inhibitors, *n* (%)**			
Bupropion	87 (2.2)	30 (2.2)	7 (1.1)
Total	87 (2.2)	30 (2.2)	7 (1.1)
**Tetracyclic piperazino-azepine, *n* (%)**			
Mirtazapine	10 (0.3)	2 (0.2)	1 (0.2)
Total	10 (0.3)	2 (0.2)	1 (0.2)
**Serotonin modulators, *n* (%)**			
Nefazodone	90 (2.2)	27 (2.0)	9 (1.4)
Trazodone	248 (6.1)	81 (5.9)	25 (3.9)
Total	338 (8.3)	108 (7.9)	34 (5.3)
**SNRIs, *n* (%)**			
Venlafaxine	497 (12.3)	140 (10.2)	53 (8.2)
Total	497 (12.3)	140 (10.2)	53 (8.2)

Percentages may not add up to 100% due to rounding. Groups are not mutually exclusive since a woman could have received more than one antidepressant.

* Number of women who received at least one antidepressant during the first, second, or third trimester, respectively.

A total of 349 women had received a diagnosis of major depressive disorder either during pregnancy or during the 12 months before pregnancy. For the following drugs, all prescriptions were at optimal dosage: bupropion, clomipramine, doxepin, desipramine, fluvoxamine, and nortriptyline (Table 3). Paroxetine was at optimal dosage in 99.5% of women and fluoxetine in 99.4% of women. In contrast, trazodone, amitriptyline, and sertraline were the antidepressants with the highest percentage of prescriptions with sub-dosage during pregnancy (40.3, 26.3, and 12.7%, respectively) (Table 3). No subjects had prescriptions that were considered overdosed according to guidelines.

**Table 3:** Dosage characteristics during the gestational period

Antidepressant	Optimal dosage*, *n* (%)	Sub-dosage*, *n* (%)	Overdosage*, *n* (%)	Range according to published guidelines^22^
Amitriptyline	28 (73.7)	10 (26.3)	0 (0.0)	25–300
Amoxapine**	—	—	—	Not available
Bupropion	89 (100.0)	0 (0.0)	0 (0.0)	100–300
Citalopram	149 (97.4)	4 (2.6)	0 (0.0)	10–60
Clomipramine	26 (100.0)	0 (0.0)	0 (0.0)	25–300
Desipramine	63 (100.0)	0 (0.0)	0 (0.0)	25–300
Doxepin	3 (100.0)	0 (0.0)	0 (0.0)	25–300
Fluoxetine	156 (99.4)	1 (0.6)	0 (0.0)	10–80
Fluvoxamine	27 (100.0)	0 (0.0)	0 (0.0)	50–300
Imipramine***	N/A	N/A	N/A	25–300
Maprotiline***	N/A	N/A	N/A	30–225
Mirtazapine**	—	—	—	Not available
Moclobemide**	—	—	—	Not available
Nefazodone	26 (96.3)	1 (3.7)	0 (0.0)	100–600
Nortriptyline	7 (100.0)	0 (0.0)	0 (0.0)	10–200
Paroxetine	373 (99.5)	2 (0.5)	0 (0.0)	10–60
Phenelzine**	—	—	—	Not available
Sertraline	199 (87.3)	29 (12.7)	0 (0.0)	50–225
Tranylcypromine**	—	—	—	Not available
Trazodone	80 (59.7)	54 (40.3)	0 (0.0)	75–600
Trimipramine***	N/A	N/A	N/A	25–300
Venlafaxine	33 (89.2)	4 (10.8)	0 (0.0)	75–225

N/A, Nonapplicable.

* The percentage of prescriptions with optimal dosage, for a given antidepressant, was calculated by dividing all prescriptions that were optimally prescribed by the total number of prescriptions. The same calculation was performed to determine the percentage of prescriptions with sub-dosage and overdosage. These percentages were restricted to women who had at least one diagnosis of major depressive disorder (ICD-9 codes: 296.2 and 296.3) in any period prior to receiving the antidepressant.

** The range for the following antidepressants were not available in published guidelines: amoxapine, mirtazapine, moclobemide, phenelzine, and tranylcypromine.

*** The following antidepressants were not prescribed in our cohort for women who had at least one diagnosis of major depressive disorder during the gestational period: imipramine, maprotiline, and trimipramine.

Among antidepressant users on the first day of gestational age (*n* = 2442), 4.7% of women switched to another class of antidepressants at least once during the first trimester. SSRIs were the class of antidepressants to which women switched to the most (40 [1.6%]), followed by the serotonin modulators (30 [1.2%]), and SNRIs (24 [1.0%]). More specifically, 6.1% of women switched to another type of antidepressant at least once during the first trimester. Paroxetine (24 [1.0%]) and venlafaxine (24 [1.0%]) were the types of antidepressants to which women switched to the most.

The results concerning predictors of antidepressant use at the beginning of pregnancy are shown in Table 4. Factors significantly associated with antidepressant use on the first day of gestation and measured on this day were older maternal age, being on welfare, and calendar year. Having received at least six different types of medications other than antidepressants, having at least two different prescribers, having a higher number of visits to the physician, and having at least one diagnosis of depression in the year prior to the first day of gestation were also significantly associated with antidepressant use on the first day of gestation. In contrast, having at least one visit to the emergency department or one hospitalisation decreased the probability of using an antidepressant on the first day of gestation.

**Table 4:** Predictors of antidepressant use on the first day of gestation

	Users on the first day of gestation (*n* = 2442)	Nonusers on the first day of gestation (*n* = 95 238)	Unadjusted OR (95% CI)	Adjusted OR (95% CI)*
**On the first day of gestation**				
Maternal age, years (mean, SD)	29.8 (6.4)	27.34 (6.1)	1.07 (1.06–1.07)	1.07 (1.06–1.07)
Urban dwellers, *n* (%)	1993 (81.6)	76 086 (79.9)	1.12 (1.01–1.24)	0.94 (0.84–1.05)
Welfare, *n* (%)	1200 (49.1)	31 808 (34.6)	1.82 (1.68–1.98)	1.41 (1.29–1.54)
**During the 12 months before the first day of gestation**				
Number of different prescribers, *n* (%)				
1	273 (11.2)	50 012 (52.5)	1.00	1.00
≥2	2169 (88.8)	45 226 (47.5)	8.78 (7.74–9.97)	3.92 (3.36–4.57)
Number of different medications used other than antidepressants, *n* (%)				
0–2	692 (28.3)	62 688 (65.8)	1.00	1.00
3–5	936 (38.3)	24 523 (25.8)	3.46 (3.13–3.82)	1.13 (1.00–1.27)
≥6	814 (33.3)	8027 (8.4)	9.19 (8.28–10.20)	1.79 (1.57–2.05)
Abortion/miscarriage, *n* (%)	45 (1.8)	2006 (2.1)	0.87 (0.65–1.18)	0.83 (0.60–1.13)
Number of visits to a physician, *n* (%)				
0–2	172 (7.0)	33 057 (34.7)	1.00	1.00
3–5	392 (16.1)	25 664 (27.0)	2.94 (2.45–3.51)	1.52 (1.26–1.83)
≥6	1878 (76.9)	36 517 (38.3)	9.88 (8.45–11.56)	2.42 (2.03–2.89)
Emergency department visit/hospitalisation, *n* (%)	482 (19.7)	13 919 (14.6)	1.44 (1.30–1.59)	0.72 (0.64–0.80)
Diagnosis of depression**, *n* (%)	1193 (48.9)	4172 (4.4)	20.85 (19.15–22.70)	11.59 (10.57–12.72)
**Calendar year on the first day of gestation, *n* (%)**				
1 January 1998 to 31 December 1998	438 (17.9)	25 267 (26.5)	1.00	1.00
1 January 1999 to 31 December 1999	519 (21.25)	22 098 (23.2)	1.36 (1.19–1.54)	1.34 (1.17–1.54)
1 January 2000 to 31 December 2000	525 (21.50)	18 568 (19.5)	1.63 (1.44–1.85)	1.59 (1.38–1.82)
1 January 2001 to 31 December 2001	550 (22.5)	16 788 (17.6)	1.89 (1.66–2.15)	1.93 (1.68–2.21)
1 January 2002 to 31 December 2002	410 (16.8)	12 517 (13.1)	1.89 (1.65–2.17)	1.86 (1.60–2.16)

Percentages may not add up to 100% due to rounding.

* Adjusted for the covariates in the table.

** ICD-9 codes: 296.x, 300.4, 309, 311.

Among women with a delivery (live birth or stillbirth), predictors of antidepressant use at the end of pregnancy were also identified (Table 5). Predictors were similar to those found at the beginning of pregnancy.

**Table 5:** Predictors of antidepressant use on the last day of gestation

	Users on the last day of gestation (*n* = 452)	Nonusers on the last day of gestation (*n* = 56 529)	Unadjusted OR (95% CI)	Adjusted OR (95% CI)*
**At the end of pregnancy**				
Maternal age, years (mean, SD)	30.2 (5.9)	28.2 (5.6)	1.06 (1.05–1.08)	1.06 (1.04–1.07)
Urban dwellers, *n* (%)	354 (78.3)	43 216 (76.5)	1.11 (0.89–1.39)	0.90 (0.71–1.15)
Welfare, *n* (%)	241 (53.3)	17 769 (32.7)	2.35 (1.96–2.83)	1.39 (1.12–1.71)
**During the 12 months before the first day of gestation**				
Number of different prescribers, *n* (%)				
1	74 (16.4)	29 960 (53.0)	1.00	1.00
≥2	378 (83.6)	26 569 (47.0)	5.76 (4.49–7.39)	1.72 (1.24–2.39)
Number of different medications use other than antidepressants, *n* (%)				
0–2	136 (30.1)	37 479 (66.3)	1.00	1.00
3–5	162 (35.8)	14 419 (25.5)	3.10 (2.46–3.89)	0.95 (0.71–1.26)
≥6	154 (34.1)	4631 (8.2)	9.16 (7.26–11.57)	1.09 (0.78–1.52)
Abortion/miscarriage, *n* (%)	7 (1.6)	864 (1.5)	1.01 (0.48–2.15)	1.10 (0.51–2.40)
Number of visits to a physician, *n* (%)				
0–2	43 (9.5)	19 602 (34.7)	1.00	1.00
3–5	78 (17.3)	15 058 (26.6)	2.36 (1.63–3.43)	1.35 (0.91–1.99)
≥6	331 (73.2)	21 869 (38.7)	6.90 (5.02–9.49)	1.66 (1.15–2.41)
Emergency department visit/hospitalisation, *n* (%)	98 (21.7)	8265 (14.6)	1.62 (1.29–2.02)	0.84 (0.65–1.08)
Diagnosis of depression**, *n* (%)	187 (41.4)	2401 (4.3)	15.91 (13.14–19.27)	4.36 (3.44–5.54)
**Between the first day of gestation and the end of pregnancy**				
Number of different prescribers, *n* (%)				
1	67 (14.8)	38 784 (68.6)	1.00	1.00
≥2	385 (85.2)	17 745 (31.4)	12.56 (9.68–16.29)	5.17 (3.79–7.06)
Number of different type of medications use other than antidepressants, *n* (%)				
0–2	163 (36.1)	44 791 (79.2)	1.00	1.00
3–5	167 (37.0)	9781 (17.3)	4.69 (3.78–5.83)	1.12 (0.87–1.45)
≥6	122 (27.0)	1957 (3.5)	17.13 (13.49–21.76)	2.12 (1.55–2.89)
Number of prenatal visits, *n* (%)				
0–5	100 (22.12)	11 222 (19.85)	1.00	1.00
6–11	239 (52.88)	31 275 (55.33)	0.86 (0.68–1.08)	0.84 (0.65–1.09)
≥12	113 (25.0)	14 032 (24.82)	0.90 (0.69–1.18)	0.89 (0.66–1.21)
Emergency department visit/hospitalisation, *n* (%)	393 (87.0)	49 808 (88.1)	0.90 (0.68–1.18)	1.04 (0.76–1.41)
Diagnosis of depression**, *n* (%)	139 (30.8)	1001 (1.8)	24.64 (19.99–30.38)	5.21 (4.02–6.74)
Gestational age				
<37 weeks	57 (12.6)	4050 (7.2)	1.00	1.00
≥37 weeks	395 (87.4)	52 479 (92.8)	0.54 (0.40–0.71)	0.77 (0.56–1.05)
**Calendar year on the first day of gestation, *n* (%)**				
1 January 1998 to 31 December 1998	66 (14.6)	14 631 (25.9)	1.00	1.00
1 January 1999 to 31 December 1999	99 (21.9)	13 329 (23.6)	1.65 (1.21–2.25)	1.66 (1.19–2.30)
1 January 2000 to 31 December 2000	101 (22.4)	11 171 (19.8)	2.00 (1.47–2.74)	1.96 (1.41–2.72)
1 January 2001 to 31 December 2001	109 (24.1)	9984 (17.7)	2.42 (1.78–3.29)	2.60 (1.88–3.61)
1 January 2002 to 31 December 2002	77 (17.0)	7414 (13.1)	2.30 (1.66–3.20)	2.36 (1.65–3.36)

Percentages may not add up to 100% due to rounding.

* Adjusted for the covariates in the table.

** ICD-9 codes: 296.x, 300.4, 309, 311.

## Discussion

To our knowledge, this is the first study to longitudinally investigate the prevalence of antidepressant use before, during, and after pregnancy. In addition, it is the first study to determine the percentage of switches and the dosages and classes of antidepressants used during pregnancy. Furthermore, predictors of antidepressant use on the first day and on the last day of gestation were identified. Antidepressant use was reduced by nearly half during the first trimester (3.7 versus 6.6%, *P* ≤ 0.01), only to increase during the postpartum period (7.0 versus 6.6%, *P* ≤ 0.01). These data confirm that healthcare providers remain cautious in prescribing antidepressants during pregnancy or that the women themselves hesitate to take these drugs during this time period. These results are consistent with a recent study, which found that the prescription rates of most medications used to treat chronic diseases decrease during pregnancy, especially those for antidepressants.^24^

The three most prevalent classes of antidepressants prescribed during pregnancy were SSRIs (64.4%), SNRIs (12.3%), and tricyclics (12.1%). The use of MAOIs was limited (0.1%). Although until now no definitive data were available, it has been assumed that the use of antidepressants during pregnancy reflects usage patterns in the general population.^25^ Indeed, in the general population, SSRIs have been the most widely used antidepressant because of their established efficacy, their milder adverse effect profile, and their safety in overdose.^25^ In certain circumstances, agents such as venlafaxine, bupropion, and mirtazapine, as well as tricyclic antidepressants, have been preferred by healthcare providers. In contrast, the safety concerns regarding drug and food interactions have limited the use of MAOIs.^25^ Our results seem to confirm these findings during pregnancy. However, this utilisation pattern may soon change, given the recent warnings on gestational use of paroxetine and negative outcomes on the newborn.^26^,^27^

Our data indicate that antidepressants are prescribed according to published recommendations 92.3% of the time during pregnancy. More precisely, antidepressants are prescribed under the recommended dosage 7.7% of the time and never over the recommended dosage during the gestational period. However, changes in maternal physiology during pregnancy are important. Higher dose requirements for most antidepressants are necessary to avoid subtherapeutic blood concentrations.^28^

Only 6.1% of women switched from one antidepressant to another during the first trimester, whereas the majority of women discontinued their current antidepressant. It would have been expected that more women would have switched from one antidepressant to another instead of discontinuing their treatment. This fact reinforces the idea that physicians or women prefer terminating an antidepressant treatment rather than selecting another one with a better safety profile. Instead, careful treatment planning should be in place for those women on antidepressants who plan to conceive or who become pregnant.

We found several predictors using an antidepressant at the beginning and at the end of pregnancy. Both models indicated that a higher number of prescribers before and during pregnancy, a higher number of visits to the physician before pregnancy, and having a diagnosis of depression before or during pregnancy increased the probability of using antidepressants on the first day and on the last day of gestation. These results suggest that subjects who initiated or continued receiving antidepressants at these times were those women who were likely to be less healthy than those who did not initiate or discontinued using them. In addition, having a diagnosis of depression was associated with receiving an antidepressant, indicating that antidepressants are more likely used for the indication for which they were intended. We found that 48.9% of women had received a diagnosis of depression in the year prior to the first day of gestation. This probably represents an underestimation since diagnosis could have been made before the study period. We also found in both models that being an adherent to the RAMQ drug plan (versus a welfare recipient) decreased the likelihood of receiving an antidepressant. This could partly be explained by the fact that welfare recipients, who are of lower socio-economic level compared with adherents, are more likely to have depression.^29^ Indeed, Murphy *et al.*^29^ have found an association between socio-economic status and depression.

This study included a large sample of pregnant women, and thus, we were able to study a wide variety of characteristics in women that could predict antidepressant use before and during pregnancy. Administrative databases have the great potential of providing accurate drug dispensing history throughout pregnancy and are not affected by recall bias. Such databases give full details on the names, doses, and quantities of medications dispensed thus offering information that is almost impossible to obtain by questioning women who have to recall their use of medications over an extended period of time.^30^–^33^

The use of administrative databases has some limitations. Data are not available on women who do not use medical services during pregnancy or who give birth in a setting outside the hospital. However, given the free universal healthcare system in place in Quebec, we expect the effect of these women on our results to be minimal. In addition, data are not available on medications dispensed over-the-counter without a prescription. This was not problematic in the present study because all antidepressants require a written prescription. An unresolved problem is that we do not have data on the number of women who discontinued their antidepressant therapy to initiate herbal treatments for depression, such as St John's Wort.

The prevalence of antidepressant use was calculated on the basis of the drugs dispensed to study subjects and does not reflect the actual intake. However, our study has the advantage over field studies, which usually rely on self-reported drug histories and therefore are prone to recall bias.

In the calculation of optimal dosage, sub-dosage, and overdosage, we used guidelines for treating depression during pregnancy published in 2003. Our cohort spanned a 5-year period, 1998–2002, and thus, we applied 2003 guidelines to earlier years when official recommendations on how to prescribe antidepressants during pregnancy were not available. Despite the limitation of using the 2003 guidelines, we were able to determine how appropriately physicians prescribed antidepressants during pregnancy in the absence of guidelines. In fact, it should be noted that the 2003 guidelines were based on publications that were available in the years prior or during the time period of this study. We found that physicians tended to prescribe suboptimal dosages during pregnancy, which may be related to the fear of potential negative effects on the fetus.

The RAMQ database offers information on planned abortions and miscarriages, but it was not always easy to distinguish between them. This may have resulted in misclassification, which could explain the low percentage of miscarriages (4.8%). Finally, drugs dispensed during hospitalisations are not included in the RAMQ database. As such, it could explain why having a hospitalisation decreased the likelihood of using an antidepressant on the first day of gestation.

The RAMQ database provides information on welfare recipients and on adherents of the RAMQ drug plan only and not on individuals who are covered by private drug insurance. As such, socio-economic status may act as an effect modifier. That is, these women may be more likely to use antidepressants than those covered by private insurance programmes. This may limit the generalisation of our study, but we feel that it does not invalidate the results.

## Conclusion

The results of this study confirm that either women avoid taking antidepressants during pregnancy or physicians hesitate prescribing them for fear of harming the fetus. Much research has been conducted to determine the negative impact of antidepressants during pregnancy. However, it is important to assess the impact of not treating depressive symptoms and the consequences that may result on the mother and on the newborn. As such, more studies are needed to evaluate the impact of the decreased use of antidepressants during pregnancy.
